# Homogenisation for the monodomain model in the presence of microscopic fibrotic structures

**DOI:** 10.1016/j.cnsns.2022.106794

**Published:** 2023-01

**Authors:** Brodie A.J. Lawson, Rodrigo Weber dos Santos, Ian W. Turner, Alfonso Bueno-Orovio, Pamela Burrage, Kevin Burrage

**Affiliations:** a Centre for Data Science, Queensland University of Technology, 2 George Street, Brisbane, 4000, Queensland, Australia; b ARC Centre of Excellence for Mathematical and Statistical Frontiers, Queensland University of Technology, 2 George Street, Brisbane, 4000, Queensland, Australia; c School of Mathematical Sciences, Queensland University of Technology, 2 George Street, Brisbane, 4000, Queensland, Australia; d Graduate Program on Computational Modelling, Universidade de Federal de Juiz de Fora, Rua Jose Lourenco Kelmer s/n, Juiz de Fora, 36036-900, Minas Gerais, Brazil; e Department of Computer Science, University of Oxford, Parks Rd, Oxford, OX1 3QD, Oxfordshire, United Kingdom

**Keywords:** 35B27, 74Q10, Homogenisation, Cardiac fibrosis, Cardiac electrophysiology, Monodomain model, Block homogenisation, Volume averaging

## Abstract

Computational models in cardiac electrophysiology are notorious for long runtimes, restricting the numbers of nodes and mesh elements in the numerical discretisations used for their solution. This makes it particularly challenging to incorporate structural heterogeneities on small spatial scales, preventing a full understanding of the critical arrhythmogenic effects of conditions such as cardiac fibrosis. In this work, we explore the technique of homogenisation by volume averaging for the inclusion of non-conductive micro-structures into larger-scale cardiac meshes with minor computational overhead. Importantly, our approach is not restricted to periodic patterns, enabling homogenised models to represent, for example, the intricate patterns of collagen deposition present in different types of fibrosis. We first highlight the importance of appropriate boundary condition choice for the closure problems that define the parameters of homogenised models. Then, we demonstrate the technique’s ability to correctly upscale the effects of fibrotic patterns with a spatial resolution of 10µm into much larger numerical mesh sizes of 100-250µm. The homogenised models using these coarser meshes correctly predict critical pro-arrhythmic effects of fibrosis, including slowed conduction, source/sink mismatch, and stabilisation of re-entrant activation patterns. As such, this approach to homogenisation represents a significant step towards whole organ simulations that unravel the effects of microscopic cardiac tissue heterogeneities.

## Highlights

• Upscaling for feasible simulation of microscale fibrosis in cardiac electrophysiology
• Practical consideration of upscaling for dynamics of sharp-fronted travelling waves
• Demonstrated recovery of pro-arrhythmic effects of fibrosis in upscaled models
• Selection of boundary conditions to balance numerical and upscaling errors
• Significant speed-up for monodomain model simulation in the presence of microfibrosis

## 1 Introduction

Computational simulation plays a critical role in our understanding of the functioning of the heart, in particular the complex manifestations of its excitable media dynamics into dangerous arrhythmias [1]. Cardiac fibrosis, the pathological formation of scar tissue in the heart [2], is an important contributor to many types of arrhythmias. Fibrosis’ arrhythmogenic impacts depend on its spatial organisation on both microscopic [3] and macroscopic [4] scales. However, owing both to limitations of computational feasibility and the resolution of clinical imaging approaches, anatomically-accurate meshes used in the simulation of electrical signalling in the heart typically have spacings of a minimum of 100 micrometres [5]. This is at least an order of magnitude too large to resolve the complex and varied microscopic structures of fibroblast-deposited collagen that interfere with wave propagation [6]. It is therefore vital that such “sub-mesh scale” effects of fibrotic obstacles be incorporated into simulations without altering the mesh spacing. Upscaling these small-scale effects into a larger-scale mesh also represents a significant computational time saving more generally, and one that may be used alongside other acceleration approaches such as improved numerical techniques [7], [8] or advanced hardware architectures [9].

The incorporation of small-scale fibrotic structures into larger-scale cardiac electrophysiology simulations has seen some consideration. Through a clever node re-labelling, Costa et al. were able to incorporate disconnections between neighbouring elements due to strands of collagen [10], although such an approach does not necessarily account for the effects of obstacles *within* mesh elements. An alternative approach is based on the mathematical technique of *homogenisation*, which explicitly seeks to represent micro-scale effects as modifications to macro-scale problems [11]. In cardiac electrophysiology, homogenisation can be used to derive the well-known bidomain model [8], [12], [13], [14], [15], [16], as well as its modification for less ordered arrangements of cells [17] or non-Ohmic tissue conduction [18]. Homogenisation for the inclusion of fibrotic obstructions has been almost solely limited to spatially periodic structures [19], [20]. Austin et al. [21] used multigrid-based homogenisation to upscale arbitrarily arranged obstacles, but only considered performance in terms of activation maps. Some of the authors have also recently explored an alternative homogenisation using the eikonal approximation [22], [23]. However, none of these works have considered how well homogenised cardiac electrophysiology models can capture the mechanisms through which microscopic obstacles can act as arrhythmia precursors, arguably the primary aspect of interest.

In this work, we use a volume averaging approach for the incorporation of arbitrary structures of microscopic obstacles into a larger-scale problem. In contrast to the above approaches towards upscaling, volume averaging naturally derives homogenised models that modify more than just the conductivity tensor, improving the capture of dynamics such as wave die-out due to electrotonic loss. We also explore the issue of boundary condition choice for homogenisation sub-problems in terms of practical performance in this challenging homogenisation context, involving both sharp-fronted travelling wave dynamics and completely non-conductive regions. Finally, we demonstrate successful capture of several important pro-arrhythmic effects of cardiac fibrosis by block homogenised models with at least one order of magnitude fewer nodes than the corresponding fine-scale models.

Our homogenisation approach represents a significant advance over other upscaling techniques that have been presented for cardiac electrophysiology, owing to its ability to capture the effects of heterogeneity in excitable tissue and to deal with non-periodic patterns of obstruction. The formulation we present is applicable to all types of meshes and any number of dimensions, and we provide full implementation details and MATLAB code for the case of two-dimensional regular grids. The approach represents an important step towards full-scale (chamber or organ level) simulations that respect the fine-scale arrangement of fibrotic obstacles, such as those seen in histological sections [6] or obtained through recently demonstrated techniques for computational generation of realistic patterning of fibrosis [24], [25].

## 2 Methodology

### 2.1 The monodomain model in fibrotic myocardium

The dynamics of cardiac excitation are governed by the monodomain model [7], a simplification of the bidomain model that offers similar quality of predictions in many contexts [26], [27], [28]. The monodomain model is a reaction–diffusion partial differential equation, coupled to a set of ordinary differential equations that describe the behaviour of the reaction term. In the presence of non-conductive obstacles, the monodomain model may be expressed in the form

(1) $$\frac{\partial v}{\partial t}=\nabla \cdot \left(D\nabla v\right)-\frac{1}{C_{m}}\left(I_{\mathrm{ion}}\left(v,s\right)+I_{\mathrm{stim}}\right)\;\text{within conducting tissue}$$

$$\frac{ds}{dt}=f\left(v,s\right)\;\text{within conducting tissue}$$

$$0\;=\left(D\nabla v\right)\cdot \overset{ˆ}{n}\;\text{on boundaries (incl. obstructions)}.$$

Here v is the membrane potential (lower case used to denote a micro-scale variable), $C_{m}$ the membrane capacitance and D the conductivity tensor. $I_{\mathrm{stim}}$ refers to externally supplied stimulus current, and $I_{\mathrm{ion}}$ describes the flow of ions in/out of cardiac cells, which depends on both the membrane potential and a set of state variables s.

There are many different models describing the voltage-dependent nature of cell ion channels, to varying levels of biophysical detail, that provide a definition for $I_{\mathrm{ion}}$ and f [29]. Broadly, however, $I_{\mathrm{ion}}$ produces relaxation oscillation behaviour (such as that of the well-known Fitzhugh–Nagumo model [7]) that results in sharp-fronted travelling waves in conductive media. Here, our simulations use the reduced version of the ten Tusscher et al. ionic model presented in [30]. This model represents action potentials in human ventricular epicardium with formulations for all of the major Na${}^{+}$, K${}^{+}$, Ca^2＋^ channels, but fixes several ion concentrations and makes quasi-steady-state assumptions for rapidly varying gating variables in order to significantly reduce computational cost.

**Fig. 1:**
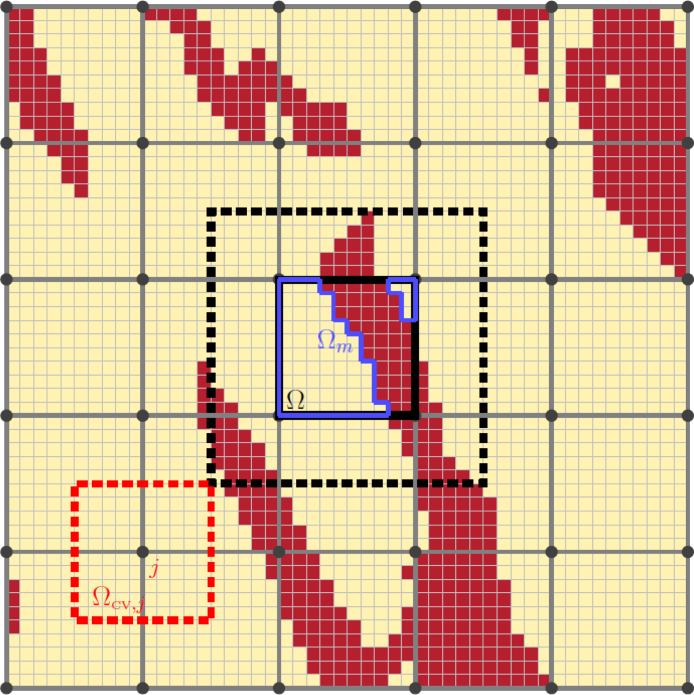
The mesh and sub-mesh involved with homogenisation. An example pattern of collagenous obstructions due to fibrosis (dark red) defined on a small-scale mesh, and the larger mesh on which effective conductivity tensors are defined. A single averaging volume (Ω) is marked in black, along with its associated conductive region, $\Omega_{m}$, in blue. The black dotted line shows the region on which the closure problem associated with Ω is solved when a layer of skin is included around the averaging volume (see Methodology). An example control volume used by the finite volume approach used for numerical discretisation is also pictured, in red.

We take the common approach of representing fibrosis-afflicted tissue as a combination of conductive myocardium and non-conductive, collagenous material deposited by fibroblasts [31], [32]. These fibrotic obstructions are defined on a fine-scale grid of spacing Δx=10µm, a similar order to the pixels in histological images indicating the spatial arrangement of collagenous obstacles in cardiac fibrosis [6]. The homogenised models we construct represent the effects of these obstacles on a regular, coarser grid as visualised in Fig. 1. For simplicity, our homogenised models use regular grids with the edges of mesh elements aligned with the finescale grid, although the theory presented later does not depend on these choices.

### 2.2 A volume-averaged monodomain model

Collagenous obstructions are found in a variety of spatial arrangements in cardiac fibrosis [6] that vary throughout the tissue [33]. As such, we are unable to define an appropriate representative volume element, and instead turn to block homogenisation [34]. Block homogenisation calculates effective properties separately for each element of the large-scale problem, as we now describe.

We derive homogenised models via the method of volume averaging, detailed in [35]. Volume averaging, in the case where non-conductive material is present, makes use of a pair of *averaging operators* that average a quantity over the conductive myocyte portion, $\Omega_{m}$, of an averaging volume, Ω (Fig. 1). The two averaging operators are

$$\langle \cdot \rangle =\frac{1}{\left|\Omega_{m}\right|}\int_{\Omega_{m}}\cdot \;d\Omega_{m},\text{intrinsic average}$$

$${\langle \cdot \rangle }_{\sup}=\frac{1}{\left|\Omega \right|}\int_{\Omega_{m}}\cdot \;d\Omega_{m},\text{superficial average},$$

and are thus linked by the volume fraction of conductive material, $\phi =\left|\Omega_{m}\right|/\left|\Omega \right|$, as

$${\langle \cdot \rangle }_{\sup}=\phi \langle \cdot \rangle .$$

The intrinsic average is practically appealing, as its value is calculated only using the conductive material and is thus unaffected by the proportion of collagen in the averaging volume. On the other hand, the superficial average does not possess this property but is mathematically useful as the averaging volume retains consistent size and shape through space. We present a brief, simplified derivation of how these operators can be used to formulate a homogenised version of (1).

Taking the superficial average of the spatially-varying equation in (1), and defining $J\left(v,s\right)=\left(I_{\mathrm{ion}}\left(v,s\right)+I_{\mathrm{stim}}\right)/C_{m}$ to simplify notation, we have

(2) $${\langle \frac{\partial v}{\partial t}\rangle }_{\sup}={\langle \nabla \cdot \left(D\nabla v\right)\rangle }_{\sup}-{\langle J\left(v,s\right)\rangle }_{\sup}.$$

Treating the obstacles as static in time, we may move the averaging operator inside the time derivative. Via the spatial averaging theorem [35, Ch. 1] we may also move the averaging operator inside the divergence operator by including a correction term. This gives

$$\frac{\partial {\langle v\rangle }_{\sup}}{\partial t}=\nabla \cdot {\langle D\nabla v\rangle }_{\sup}+\frac{1}{\left|\Omega \right|}\int_{\sigma_{m}}\left(D\nabla v\right)\cdot \overset{ˆ}{n}\;d\sigma_{m}-{\langle J\left(v,s\right)\rangle }_{\sup}$$

where $\sigma_{m}$ denotes the surface of the interface between the conductive and non-conducting regions. Due to the no-flux boundary condition on obstacles, this integral correction term in fact goes to zero, but carefully applying the spatial averaging theorem is necessary as this is what necessitates the use of the superficial average and ensures the volume fraction, ϕ, features correctly in the homogenised model. Converting now to the intrinsic average, we obtain

(3) $$\phi \frac{\partial \langle v\rangle }{\partial t}=\nabla \cdot \left(\phi \langle D\nabla v\rangle \right)-\phi \langle J\left(v,s\right)\rangle .$$

We wish to express (3) solely in terms of a macroscopic variable, V=〈v〉, which we can achieve by choosing to define the effective conductivity tensor such that

(4) $$D_{\mathrm{eff}}\nabla V=\langle D\nabla v\rangle ,$$

and making the simplifying approximation

(5) 〈J(v,s)〉≈J(V,s).

This approximation may seem questionable in this context, where J(v,s) is a strongly nonlinear function and the sharp fronts of travelling waves mean that v can also vary significantly over some averaging volumes. However, we point out that any increase of mesh spacing in solving the monodomain equation (that is, independent of any homogenisation) implicitly makes a similar approximation in that it too replaces finer-scale dynamics with some smoothed representation at lower resolution. We also choose our numerical scheme to minimise the impact of this approximation, discussed subsequently.

Substituting Eqs. (4), (5) into (3) gives the homogenised monodomain model describing the large-scale behaviour of the system,

(6) $$\phi \frac{\partial V}{\partial t}=\nabla \cdot \left(\phi D_{\mathrm{eff}}\nabla V\right)-\phi J\left(V,s\right)\;\text{within tissue}$$

$$\frac{ds}{dt}=f\left(V,s\right)\;\text{within tissue}$$

$$0=\left(D_{\mathrm{eff}}\nabla V\right)\cdot \overset{ˆ}{n}\;\text{on problem boundaries}.$$

Note that obstacles no longer act through the boundary conditions, but instead through their effect on $D_{\mathrm{eff}}$ and ϕ.

We briefly note that a separate technique, the smoothed boundary method, also shifts such boundary conditions into the governing equation to arrive at the formulation (6) [36], [37]. Volume averaging theory thus serves as a robust means of deriving the smoothed boundary approach. The key difference between the two approaches is their use cases. Smoothed boundary methods use especially fine grids at boundaries in order to accurately resolve their effects, whereas homogenisation by volume averaging instead seeks to represent these effects only on a larger scale, reducing computational demand.

### 2.3 Determination of effective conductivities

Eq. (4) relates the macroscopic gradient of potential ∇V to the fine-scale gradient, ∇v. These gradients will change through the course of a simulation of (1) or (6), but fixed conduction tensors can be set by instead solving separate subproblems in which a macroscopic gradient is artificially applied [38]. Imposing a macroscopic unit gradient in the ith direction ($\nabla V=e_{i}$) over the averaging volume, the ith column of the effective conductivity tensor is then given by

(7) $$D_{\mathrm{eff}}e_{i}=\langle D\nabla v_{i}\rangle ,\;\;i=1,\ldots ,d,$$

with $e_{i}$ the standard basis vectors in the d-dimensional space. Eq. (7) applies regardless of the shape of the averaging volume used, or, phrased differently, any set of d applied gradients may be used to calculate the elements of $D_{\mathrm{eff}}$ so long as they are in linearly independent directions [38].

Each of the micro-scale potential fields for different imposed gradients, $v_{i}$, are given by solving the diffusive portion of the monodomain equation on the micro-scale, (1), on the averaging volume Ω. The imposed macroscopic gradient is most naturally included by decomposing the potential fields $v_{i}$ into their macro-scale and micro-scale components,

(8) $$v_{i}=w_{i}+V_{i}\;\;\;\nabla v_{i}=\nabla w_{i}+e_{i}.$$

Then, we can solve the diffusive flow over the averaging volume in terms of $w_{i}$,

(9) $$0=\nabla \cdot \left(D\left(\nabla w_{i}+e_{i}\right)\right)\text{within conductive tissue}$$

$$0=\left(D\left(\nabla w_{i}+e_{i}\right)\right)\cdot \overset{ˆ}{n}\text{on boundaries with obstructions}.$$

These equations are typically known in the homogenisation literature as *closure* problems.

Solutions of (9) for each different elementary basis vector $e_{i}$ define the $v_{i}$’s through Eq. (8), which can then be numerically averaged to calculate the columns of the effective conductivity $D_{\mathrm{eff}}$. In some scenarios (depending on the choice of boundary conditions for (9) and the existence of disconnected islands of conductive tissue), $w_{i}$ will be defined only to within a constant, but this has no effect on the effective tensors calculated as they depend only on $\nabla w_{i}$. In our implementation, we use the minimum norm solution of the linear systems obtained by discretising (9) in these degenerate cases.

In this section, we have presented an accessible derivation of the homogenised model (6) and the closure problems (9) that define the effective conductivity values it uses through Eq. (7). In the following section, we demonstrate that under assumption of periodicity across the averaging volume, these equations may be more rigorously derived by an asymptotic scale separation approach. Readers uninterested in this derivation may skip to Section 2.5.

### 2.4 Derivation via scale separation

Here, we demonstrate how the homogenisation approach we take may be asymptotically derived via scale separation. The derivation follows [39, Ch.5], but is generalised to allow for tensor conductivity on the micro-scale and the presence of non-conductive inclusions.

The scale separation approach begins by splitting the space variable x into two parts that are then treated as independent, a macro-scale variable x and a micro-scale variable y=x/ϵ. Here ϵ≪1 is a small parameter indicating the difference in scale. Under this scale separation, the gradient operator becomes

$$\nabla \to \nabla_{x}+\frac{1}{\epsilon }\nabla_{y}.$$

We assume that the averaging volume Ω is *representative*, that is, the substructure (occlusions as well as D) is periodic outside of Ω. Under this assumption, the conductivity D depends only on y. In practice, we are only pretending this is true for the calculation of the current volume Ω’s effective conductivity, and the x-dependence is then represented by applying the approach separately to each macroscopic mesh element composing the domain.

We take an asymptotic expansion for v,

$$v=v_{0}+\epsilon v_{1}+\epsilon^{2}v_{2}+\cdots \;.$$

Substituting this and the scale-separated gradient operator into the monodomain model (1), we arrive at the following sets of equations:

Within conducting phase,

$$O\left(1\right):\;\;0=\nabla_{y}\cdot \left(D\nabla_{y}v_{0}\right)$$

$$O\left(\epsilon \right):\;\;0=\nabla_{x}\cdot \left(D\nabla_{y}v_{0}\right)+\nabla_{y}\cdot \left(D\left(\nabla_{x}v_{0}+\nabla_{y}v_{1}\right)\right)$$

$$O\left(\epsilon^{2}\right):\;\;\;\;\frac{\partial v_{0}}{\partial t}=\nabla_{x}\cdot \left(D\left(\nabla_{x}v_{0}+\nabla_{y}v_{1}\right)\right)+\nabla_{y}\cdot \left(D\left(\nabla_{x}v_{1}+\nabla_{y}v_{2}\right)\right)-J$$

Boundaries with non-conducting phase,

$$O\left(1\right):\;\;0=\left(D\nabla_{y}v_{0}\right)\cdot \overset{ˆ}{n}$$

$$O\left(\epsilon \right):\;\;0=\left(D\left(\nabla_{x}v_{0}+\nabla_{y}v_{1}\right)\right)\cdot \overset{ˆ}{n}$$

$$O\left(\epsilon^{2}\right):\;\;0=\left(D\left(\nabla_{x}v_{1}+\nabla_{y}v_{2}\right)\right)\cdot \overset{ˆ}{n}$$

The O(1) equations simply state that $v_{0}$ depends only on x, and is thus our macro-scale variable of interest. The O(ϵ) equation, after using $\nabla_{y}v_{0}=0$, reduces to

(10) $$\nabla_{y}\cdot \left(D\left(\nabla_{x}v_{0}+\nabla_{y}v_{1}\right)\right)=0.$$

This motivates the choice of a form for $v_{1}$,

(11) $$v_{1}=w\left(y\right)\cdot \nabla_{x}v_{0}+k\left(x\right),$$

so that the O(ϵ) equation can be satisfied independent of the form of $v_{0}$. Substituting (11) into (10) and the O(ϵ) boundary condition, we see that we must have

$$0=\nabla_{y}\cdot \left(D\left(\nabla_{y}w_{i}+e_{i}\right)\right)\text{in tissue}$$

$$0=\left(D\left(\nabla_{y}w_{i}+e_{i}\right)\right)\cdot \overset{ˆ}{n}\text{on boundaries with collagen.}$$

These are the closure problems (9) presented in the main document, where again we have reserved discussion of the boundary conditions applied to the edges of Ω for later.

The $O\left(\epsilon^{2}\right)$ equation is simplified by averaging. However, in the presence of non-conductive occlusions, we must use the superficial average over only the conductive portion of Ω, here again denoted as $\Omega_{m}$. We first consider the superficial average of the second divergence term,

$$\frac{1}{\left|\Omega \right|}\int_{\Omega_{m}}\nabla_{y}\cdot \left(D\left(\nabla_{x}v_{1}+\nabla_{y}v_{2}\right)\right)\;d\Omega_{m}.$$

This may be rewritten using the divergence theorem as a pair of surface integrals, one over the boundaries of Ω, denoted $\sigma_{b}$ and one over the internal boundaries with occlusions, denoted $\sigma_{m}$,

(12) $$\frac{1}{\left|\Omega \right|}\int_{\sigma_{m}}\left(D\left(\nabla_{x}v_{1}+\nabla_{y}v_{2}\right)\right)\cdot \overset{ˆ}{n}\;d\sigma_{m}+\frac{1}{\left|\Omega \right|}\int_{\sigma_{b}}\left(D\left(\nabla_{x}v_{1}+\nabla_{y}v_{2}\right)\right)\cdot \overset{ˆ}{n}\;d\sigma_{b}.$$

The first integral is precisely zero, due to the $O\left(\epsilon^{2}\right)$ boundary condition on occlusions. The second integral is zero due to the y-periodicity of D, $v_{1}$ and $v_{2}$.^2^

Setting that integral to zero, the averaged form of the $O\left(\epsilon^{2}\right)$ equation is

$${\langle \frac{\partial v_{0}}{\partial t}\rangle }_{\sup}={\langle \nabla_{x}\cdot \left(D\left(\nabla_{x}v_{0}+\nabla_{y}v_{1}\right)\right)\rangle }_{\sup}-{\langle J\rangle }_{\sup}.$$

This demonstrates the importance of using the superficial, as opposed to the intrinsic average, as only the former may be taken inside of the divergence operator when the substructure is not periodic (in this case, |Ω| has no dependence on x, whereas $\left|\Omega_{m}\right|$ does). Performing this reversal of order of operations, and substituting (11) for $v_{1}$, we obtain

$${\langle \frac{\partial v_{0}}{\partial t}\rangle }_{\sup}=\nabla_{x}\cdot {\langle D\left(I+\nabla w^{T}\right)\nabla_{x}v_{0}\rangle }_{\sup}-{\langle J\rangle }_{\sup},$$

with $\nabla w^{T}$ denoting an outer product (the transpose of the Jacobian of w).

Converting now to intrinsic averages and using the fact that $v_{0}$ is constant with respect to y to move it outside of the averaging operator, we obtain

$$\frac{\partial v_{0}}{\partial t}=\nabla_{x}\cdot \left(\phi \langle D\left(I+\nabla w^{T}\right)\rangle \nabla_{x}v_{0}\right)-\phi \langle J\rangle .$$

This is Eq. (6) expressed in terms of $v_{0}$ instead of V, and with the macroscopic nature of the gradient operators made explicit. The effective conductivity tensor is given by

$$D_{\mathrm{eff}}=\langle D\left(I+\nabla w^{T}\right)\rangle ,$$

which is easily seen to be equivalent to (7) after multiplying both sides by $e_{i}$.

Our approach may thus be rigorously derived via a scale separation approach, so long as the substructure is assumed to be periodic, and periodic boundary conditions are applied to the closure variables ($v_{1}$) on the (unoccluded) edges of the representative volume. Homogenisation errors are incurred both by the truncation of the perturbation series, and the assumption that the averaging volume Ω is truly representative.

### 2.5 Closure problem boundary conditions

We have not yet discussed the boundary conditions (BCs) applied on the boundaries of the averaging volume in Eq. (9). Although periodic BCs are most common and best supported by theory, in practice a variety of BCs have seen use in Laplacian homogenisation [38], [40]. As we demonstrate and further discuss in Results, the choice of BCs is non-trivial in the context of upscaling collagenous microstructures in cardiac fibrosis, and so we consider the performance of several of the most commonly seen choices here.

For a (hyper)rectangular averaging volume with side lengths $L_{i}$, these different BCs are expressed

(13) $$\text{Periodic:}\;w_{i}\left(x+L_{j}e_{j}\right)=w_{i}\left(x\right)\;\forall j\in 1,\ldots ,d$$

$$\text{Linear:}\;w_{i}=0\;x\in \sigma$$

$$\text{Confined:}\;w_{i}=0\;x\in \sigma ,e_{i}\cdot \overset{ˆ}{n}\neq 0$$

$$\;\left(\nabla w_{i}+e_{i}\right)\cdot \overset{ˆ}{n}=0\;x\in \sigma ,e_{i}\cdot \overset{ˆ}{n}=0.$$

Recalling that the closure problems calculate the resulting flow through the averaging volume due to an imposed macroscopic gradient, linear boundary conditions may be interpreted as holding all boundaries fixed according to the imposed gradient. Confined conditions, where two opposing boundaries are fixed to maintain the imposed gradient while the remaining boundaries are treated as no-flux, make Eqs. (7), (9) a numerical recreation of Darcy’s experiments that first derived hydraulic conductivity [41].

As each choice of BCs imposes one assumption or another on the microscopic flow problems defining the large-scale effective conductivities, it can be appealing to try to reduce the effects of these assumptions. One approach includes a layer of “skin” around the averaging volume, extending the domain on which (9) is solved as shown in Fig. 1 [42], [43]. Eq. (7) still only calculates averages over the averaging volume Ω, and so inclusion of skin has the effect of moving the boundaries away from the region used to calculate $D_{\mathrm{eff}}$. Extending the domain for closure problems like this also allows for periodic and confined BCs to be applied to irregularly-shaped mesh elements. The use of skin is related to the concept of “oversampling” seen in the numerical homogenisation literature [44]. Following [42], the homogenisation experiments in this work use a skin layer of width equal to half the width of the averaging volumes.

Including skin around averaging volumes incurs the loss of the guarantee that effective conductivity tensors are symmetric for linear or periodic BCs [38]. To account for this, as well as asymmetric tensors that may be derived by confined BCs, we use the algorithm of Higham [45] to find the symmetric semipositive definite tensor that is closest (in terms of Frobenius norm) to the calculated tensor. Briefly, this approach works by calculating the eigendecomposition of the symmetric portion of the initial tensor, $\left(D_{\mathrm{eff}}+D_{\mathrm{eff}}^{T}\right)/2$, setting any negative eigenvalues to zero, and then rebuilding it using this modified eigendecomposition.

### 2.6 Numerical approach

We solve the monodomain model (1) and its homogenised version (6), as well as the closure problems (9), via a cell-centred control volume finite element method. Considering Eq. (6) as an example, we integrate the transport equation over the control volume $\Omega_{\mathrm{CV}}$. Using the Gauss law and the temporally fixed nature of $\Omega_{\mathrm{CV}}$, we obtain

(14) $$\frac{\partial }{\partial t}\int_{\Omega_{\mathrm{CV}}}\phi V\;d\Omega_{\mathrm{CV}}=\int_{\sigma_{\mathrm{CV}}}\left(\phi D_{\mathrm{eff}}\nabla V\right)\cdot \overset{ˆ}{n}\;d\sigma_{\mathrm{CV}}-\int_{\Omega_{\mathrm{CV}}}\phi J\left(V,s\right)\;d\Omega_{\mathrm{CV}},$$

with $\sigma_{\mathrm{CV}}$ denoting the control volume’s surface.

To improve accuracy, in particular helping to minimise the influence of approximation (5), we evaluate these integrals by exactly integrating interpolants constructed over each mesh element using the V and J values at their vertices. Denoting the set of elements a control volume touches by $N\left(\Omega_{\mathrm{CV}}\right)$, we may write Eq. (14) as

(15) $$\frac{\partial }{\partial t}\underset{i\in N\left(\Omega_{\mathrm{CV}}\right)}{\sum }\phi_{i}\int_{\Omega_{\mathrm{CV},i}}V_{i}\;d\Omega_{\mathrm{CV},i}=\underset{i\in N\left(\Omega_{\mathrm{CV}}\right)}{\sum }\phi_{i}\left(\int_{\sigma_{\mathrm{CV},i}}\left(D_{\mathrm{eff}}\nabla V_{i}\right)\cdot \overset{ˆ}{n}\;d\sigma_{\mathrm{CV},i}-\int_{\Omega_{\mathrm{CV},i}}J_{i}\;d\Omega_{\mathrm{CV},i}\right).$$

Here subscript i refers to quantities corresponding to the ith mesh element, and V and J are the interpolations for V and J, respectively. For (bi)linear interpolants, each integrated quantity may be expressed as a linear combination of the full sets of nodal V and J values, here denoted v and j. This permits the equation for an individual control volume to be expressed

(16) $$\frac{d}{dt}\underset{i\in N\left(\Omega_{\mathrm{CV}}\right)}{\sum }\phi_{i}m_{i}^{T}v=\underset{i\in N\left(\Omega_{\mathrm{CV}}\right)}{\sum }\phi_{i}\left(k_{i}^{T}v+m_{i}^{T}j\right),$$

with the forms of $m_{i}$ and $k_{i}$ (which are defined differently for each control volume) given in Appendix A for the two-dimensional regular rectangular grids used here.

Taking the equations for each control volume together results in the finite element-like formulation

(17) $$\frac{d}{dt}Mv=Kv+Mj,$$

with mass matrix M and stiffness matrix K, the rows of which are defined by Eq. (16). This mass matrix formulation is important, as standard finite difference/volume approaches (and similarly mass lumped finite element approaches) for the monodomain model have been demonstrated to exhibit considerable error as the grid spacing increases [5].

Eq. (17) is integrated through time via the semi-implicit scheme proposed by Perego and Veneziani [46], which uses a Crank–Nicholson approach for the diffusive term and a second-order generalisation of the Rush–Larsen method for the reaction term. This takes the form

(18) $$\left(M-\frac{\Delta t}{2}K\right)v^{n+1}=\left(M+\frac{\Delta t}{2}K\right)v^{n}+M\left(\frac{3}{2}j^{n}-\frac{1}{2}j^{n-1}\right)$$

$$s_{j}^{n+1}=s_{j,\infty }^{n+\frac{1}{2}}-\left(s_{j,\infty }^{n+1/2}-s_{j}^{n}\right)\left(1-e^{-\Delta t/\tau_{j}^{n+\frac{1}{2}}}\right)$$

$$s_{j,\infty }^{n+\frac{1}{2}}=\frac{3}{2}s_{j,\infty }\left(v^{n}\right)-\frac{1}{2}s_{j,\infty }\left(v^{n-1}\right)$$

$$\tau_{j}^{n+\frac{1}{2}}=\frac{3}{2}\tau_{j}\left(v^{n}\right)-\frac{1}{2}\tau_{j}\left(v^{n-1}\right),$$

where $s_{j}$ refers to the vector of values for the jth state variable at all nodes, and $s_{j,\infty }\left(v\right)$ and $\tau_{j}\left(v\right)$ are, respectively, the functions defining the voltage-dependent steady state value and time constant of the jth state variable. The equations for $s_{j}^{n+1}$ represent exact integration of the gating variable equation

$$\frac{ds_{j}}{dt}=\frac{s_{j,\infty }\left(V\right)-s_{j}}{\tau_{j}\left(V\right)}$$

after using a second-order Adams–Bashforth type approximation to set $s_{j,\infty }$ and $\tau_{j}$ at constant values for the timestep (all state variables are gating-type variables in the model used here). We choose a timestep Δt=0.05ms.

The sensitivity of the monodomain model to the spatial discretisation poses a serious issue for both the evaluation and utilisation of homogenisation, as converting to a homogenised large-scale problem of course incurs a significant change in node spacing. As such, we further correct for the effect of the grid spacing by multiplying all conductivity tensors in our homogenised problems by a constant, such that the conduction velocity in a one-dimensional (non-fibrotic) fibre is consistent with that predicted using the finescale grid spacing (10µm). This correction factor is selected before, and entirely independent from, the homogenisation process and thus does not act to inflate the perceived performance of the homogenisation itself. Indeed, owing to the extreme sensitivity of cardiac electrophysiology models to the mesh spacing used, conductivity tuning to attain physiological conduction values on a given mesh is common practise (for example [4], [47]).

## 3 Results

### 3.1 Appropriate boundary condition selection is critical for upscaling of cardiac fibrosis

**Fig. 2:**
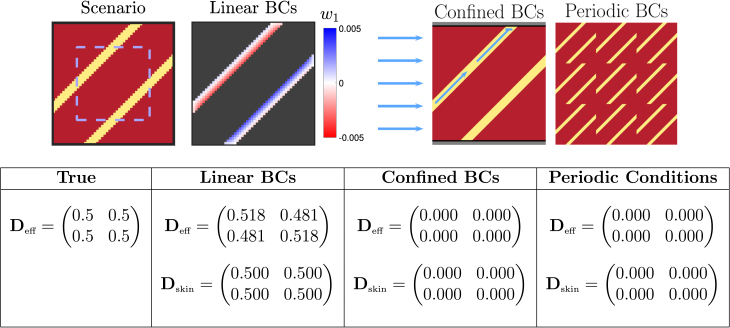
Performance of different homogenisation boundary conditions (BCs) for an averaging volume with only thin channels of conductive tissue. Conductive tissue is shown in yellow, and fibrotic obstruction in dark red (dark grey in the visualisation of the closure problem solution). Pictured is the solution to the closure problem (9) for the variable $w_{1}$, and schematic diagrams that explain the failure of the other types of boundary conditions in this scenario. The solution to the closure problem with linear boundary conditions exhibits clear patterning, with some disruption at the boundaries. The effective conductivity $D_{\mathrm{eff}}$ is found, but only approximately, due to this disruption. With confined boundary conditions, no flow can pass from left to right when the top and bottom boundaries are blocked (and analogously for when a vertical gradient is imposed), and the calculated tensor is the zero tensor. For periodic conditions, a zero tensor is obtained because the periodic extension of the pattern is seen to be a non-conducting structure. The effects of skin are considered by calculating $D_{\mathrm{eff}}$ for only the dashed blue rectangle (but still solving the closure problem over the full domain). This allows linear boundary conditions to attain $D_{\mathrm{eff}}$ correct to three decimal places, as the disruption at the boundaries has no significant effect inside the region of integration.

Consisting of non-conductive collagenous regions occurring in a variety of patterns of deposition, cardiac fibrosis is challenging to represent using upscaled tensors. For example, isthmuses through regions of scarring are of particular interest as potential substrates for arrhythmia [48], here represented on the fine scale by a thin channel(s) of conductive material passing through an otherwise non-conductive domain. However, where the ends of such channels do not align with each other at the edges of the upscaled element, the periodic assumptions underlying homogenisation then incorrectly imply a non-conductive structure [11] (see also Fig. 2 for a visual demonstration). On the other hand, where collagen is deposited in thin strands along fibres as per interstitial fibrosis [6], it is important that these strands correctly restrict conduction in the transverse direction no matter how thin they may be. As we now explore, these two scenarios represent extreme cases that demonstrate separate challenges for homogenisation in this context. As correct values for the upscaled tensors are available, and the tensors produced by the different BCs in the two scenarios are sufficiently demonstrative, we do not yet simulate any cardiac activity.

#### Conductive channels through scar tissue

We represent this scenario using a pair of thin, diagonally-oriented channels placed in otherwise non-conductive (scarred) tissue, as pictured in Fig. 2. This configuration provides a known expression for the effective conductivity tensor. Specifically, as flow is unimpeded in the direction of the channel and zero in the perpendicular direction, rotational arguments then give

(19) $$D_{\mathrm{eff}\;\left(\text{true}\right)}=\left(\begin{array}{cc} \cos\theta & -\sin\theta \\ \sin\theta & \cos\theta \end{array}\right)\left(\begin{array}{cc} D & 0\\ 0 & 0 \end{array}\right)\left(\begin{array}{cc} \cos\theta & \sin\theta \\ -\sin\theta & \cos\theta \end{array}\right)=D\left(\begin{array}{cc} \cos^{2}\theta & \cos\theta \sin\theta \\ \cos\theta \sin\theta & \sin^{2}\theta \end{array}\right).$$

We note that as we take an intrinsic formulation, the width of the channel does not appear in this result (instead represented implicitly by the value of ϕ in Eq. (6)). In a sense, the effect of obstructions on the macroscopic *amount* of possible transport is controlled by ϕ, while $D_{\mathrm{eff}}$ describes the effect of obstructions on the *character* of the transport.

In Fig. 2, we demonstrate how the different choices of BCs for Eq. (9) behave in terms of the upscaled tensor they produce for a section of tissue in this thin channel scenario. The issues with periodic and confined BCs for this scenario are immediately observed from their corresponding schematic diagrams. As the channel starts and ends do not perfectly align, the periodically extended structure is non-conductive and Eq. (9) then predicts zero effective conductivity, accordingly. Meanwhile, the nature of confined BCs allows them to identify only that flow which passes through opposing boundaries of the averaging volume. As the schematic demonstrates, diagonal channels that do not connect opposing boundaries result again in the macroscopic element being incorrectly treated as non-conductive.

In contrast, linear BCs represent the propagation of signal through conductive isthmuses quite well, returning a result for this test problem that matches approximately with the desired $D_{\mathrm{eff}}$ given by Eq. (19). The reasons for discrepancy are revealed by visualising the solution for w of one of the closure problems under these BCs (Fig. 2). The condition w=0 on the boundaries disrupts the pattern otherwise taken by w that produces the correct tensor when averaged. Including “skin” around the averaging volume, as discussed in Methodology, successfully shifts these boundary effects to outside of the region used to calculate $D_{\mathrm{eff}}$, at which point it then matches to three decimal places.

#### Fibrous collagen strands

**Fig. 3:**
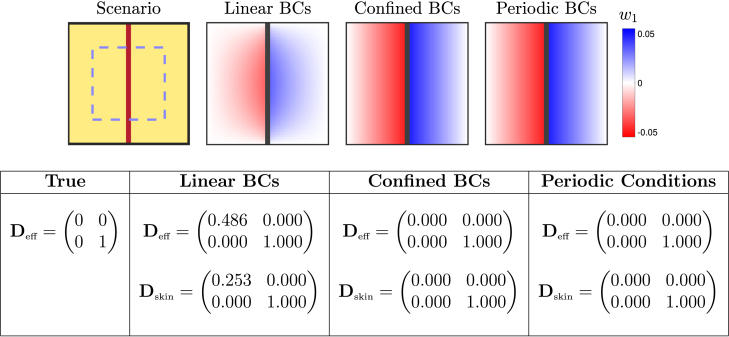
Performance of different homogenisation boundary conditions (BCs) for an averaging volume occupied by a thin strand of fibrotic obstruction. Conductive tissue is shown in yellow, and fibrotic obstruction in dark red (dark grey in visualisations of the closure problem solution). Pictured are the solutions to the closure subproblem (9) for the variable $w_{1}$, which when averaged together with the $w_{2}$ solutions (not pictured) result in the conductivity tensors given in the table. Confined and periodic conditions result in a constant gradient solution, such that $\langle D\left(e_{1}+\nabla w_{1}\right)\rangle =0$ and zero horizontal flow is correctly predicted. Linear boundary conditions result in a degradation of that solution near the boundaries, and this results in an effective tensor that still permits some horizontal flow. The effects of skin are considered by calculating $D_{\mathrm{eff}}$ for only the dashed blue rectangle (but still solving the closure problem over the full domain). Use of skin to shift averaging away from the boundaries helps reduce this effect but falls far short of eliminating it.

Thin strands of fibrotic obstruction, oriented with cardiac fibres, arise in interstitial fibrosis (also known as reactive fibrosis) [49]. Characterised by fibrosis between cells, as opposed to replacing damaged cells, interstitial fibrosis disconnects neighbouring cells and has been represented macroscopically in computational studies by similarly disconnecting neighbouring mesh elements [10]. Here, we consider this scenario by placing a thin strand of collagenous obstruction along the length of a single large-scale element, as pictured in Fig. 3.

As with the previous case, if all conductive tissue is treated as isotropically conductive with conductivity D, the effective conductivity for the element is known. The vertical band of collagen blocks horizontal propagation across the element, and vertical propagation is unaffected, giving

$$D_{\mathrm{eff}\;\left(\text{true}\right)}=\left(\begin{array}{cc} 0 & 0\\ 0 & D \end{array}\right).$$

Again, under the intrinsic definition of conductivity used here the width of the fibrotic strand affects only the volume fraction ϕ and does not appear in this result.

In Fig. 3 we present $D_{\mathrm{eff}}$ as predicted by the different choices of boundary conditions, with and without including a layer of skin. Also presented are the corresponding solutions of (9) when the macroscopic gradient is imposed in the x direction ($w_{1}$). Confined and periodic conditions produce $w_{1}$ solutions with a constant gradient from left to right, and this gradient has the appropriate magnitude such that the quantity $\langle D\nabla v_{1}\rangle =\langle D\left(\nabla w_{1}+e_{1}\right)\rangle =0$. Hence, (7) results in the correct conductivity tensor, with zero macroscopic flow in the horizontal direction.

On the other hand, when linear boundary conditions fix $w_{1}=0$ along all boundaries, the constant gradient solution is lost (Fig. 3). This results in an incorrect effective conductivity tensor that permits considerable flow in the horizontal direction. By avoiding averaging over the boundaries where the solution is most degraded, including skin has a significant positive effect on the conductivity tensor calculated using linear boundary conditions. Even still, a considerable amount of horizontal flow is permitted, and the effect of the vertical barrier on conduction is essentially lost.

Together with the results from the previous section regarding thin barriers, we see that when the arrangement of non-conducting obstacles is arbitrary, there exist scenarios for which each type of boundary condition we have considered produces a poor estimate of macroscopic conductivity. As such, we cannot select a consistently superior choice and instead now consider their performance for practical use of homogenisation in the context of cardiac electrophysiology. Specifically, we explore the potential of homogenised models to capture several key pro-arrhythmic effects of cardiac fibrosis, as represented by the presence of non-conductive fibrotic deposits.

### 3.2 Homogenised monodomain models capture macroscopic excitation propagation in obstructed tissue

Fibrotic obstructions slow the propagation of cardiac excitation through afflicted tissue, a key component of fibrosis’ pro-arrhythmic effect [50] as it decreases the “wavelength” that governs the survival of dangerous re-entries [51]. We therefore use the wavespeed through obstructed tissue as the first test of our homogenisation approach, specifically two-dimensional slices of cardiac tissue measuring 5 cm × 0.5 cm with an anisotropic conductivity tensor,

$$D=\left[\begin{array}{cc} 3 & 0\\ 0 & 1 \end{array}\right].$$

**Fig. 4:**
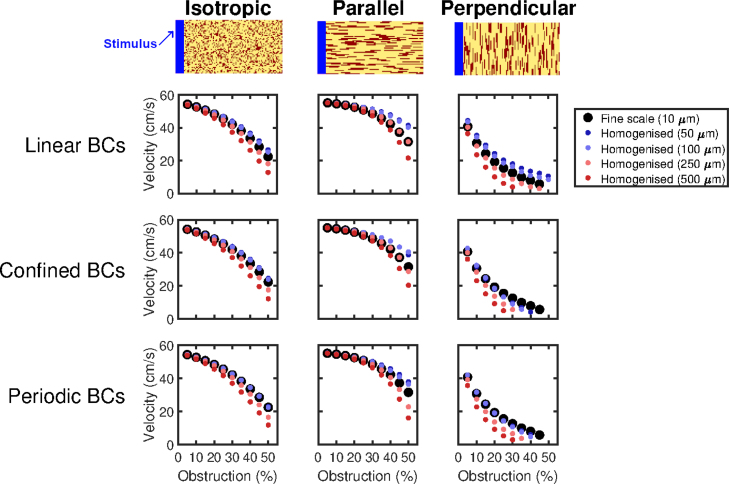
Prediction by homogenised models of conduction speeds in different types of obstructed tissue. **Top Row:** Representative sections of the 5 cm × 0.5 cm tissue slices showing the patterns of obstruction considered (obstacles in red). Pictured examples are the case of 25% obstacles. Waves of excitation move from left to right. **Bottom Rows:** Performance of the different types of boundary conditions on the three types of obstruction, for different choices of averaging volume size and boundary conditions for closure subproblems (9). Where a dot does not appear for a given level of obstruction, this corresponds to a failure to propagate the length of the tissue. Both boundary condition selection and averaging volume size have an important effect on homogenisation performance, with periodic conditions most accurate for smaller averaging volumes (Δx≤100µm) and linear conditions most accurate for the larger (Δx=250µm). Homogenised models with Δx=500µm are universally poor for the more challenging, highly obstructed problems. Best overall performance is obtained by using linear boundary conditions and a 25×25 averaging volume (10 µm up to 250 µm). Particularly notable is the case of “perpendicular” obstructions, where homogenised models using boundary conditions other than linear are prone to over-predicting conduction block.

These conductivity values are taken from [7] and reflect the faster conduction along cardiac fibres in the heart. Conduction velocities in barely-fibrotic tissue are about 55 cm s^−1^ (Fig. 4), a physiologically reasonable value for ventricular tissue. In each tissue slice, we place non-conductive obstructions at random, either 10 µm×10 µm or 90 µm×10 µm in size, with the latter oriented both parallel and perpendicular to the direction of propagation (depicted in Fig. 4). The effect of these three types of fibrosis on conduction has also been recently considered, separate from the context of homogenisation [52].

Wavespeeds predicted by the homogenised models match well with fine-scale wavespeeds, but begin to deviate as the amount of fibrotic obstruction increases and paths of conduction become more torturous (Fig. 4). The performance of different choices of boundary conditions for closure subproblems is comparable, with the superior choice also depending on the size of the averaging volume used. This is a result of the interaction between the error due to the homogenisation, and the numerical consequences of changing the grid spacing (which is exacerbated by the smaller conductivity tensors in highly-obstructed tissue). Error is consistently worst for the largest averaging volumes (Δx=500µm), the case in which homogenisation error is expected to be lowest as the ratio between characteristic length scales grows smaller [35]. This implies that the effect of changing the grid spacing is the predominant source of error. We also note that wavespeed does serve as an appropriate means of comparing the finescale and homogenised models, as this measure effectively summarises almost all of the error due to upscaling (see example activation maps given in Figure S1).

Perpendicularly aligned fibrosis presents a particularly interesting scenario, as it results in significant reduction in velocity even for small amounts of obstruction, eventually culminating in complete block of conduction when the proportion of obstructive material reaches 50%. Homogenised models will only be able to predict this block if they feature conductivities small enough to halt conduction, as all path information is lost. The results for this perpendicular fibrosis case mirror those seen in the diagonal channel example we consider in detail (Fig. 2). Specifically, homogenised models attained using linear boundary conditions can potentially over-predict successful conduction, as a connection between any averaging volume boundaries will result in a weakly conductive element (Fig. 3) even when the fine-scale structure in fact creates a dead end. On the other hand, confined and periodic boundary conditions result in homogenised models that significantly over-predict conduction block as the homogenisation process can only “see” conductive paths that fit with the assumptions that underlie them. Overall, best performance is seen using linear boundary conditions and Δx=250µm, a choice that performs very well across all of the different patternings of fibrosis tested.

**Fig. 5:**
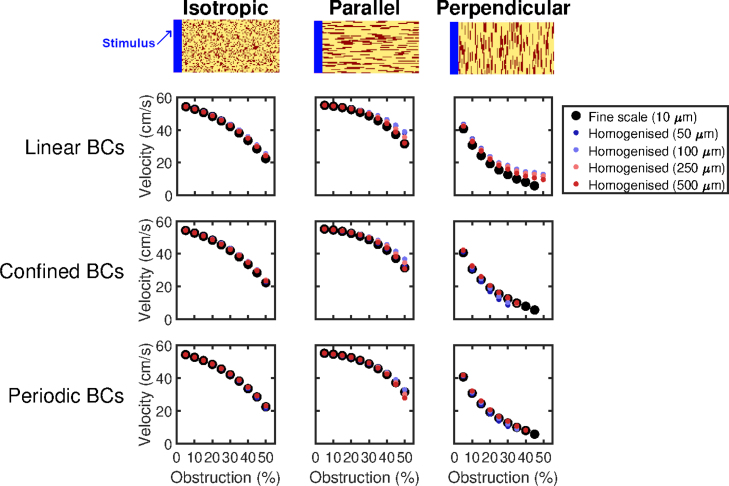
Performance of homogenisation for propagation through obstructed tissue when separated from numerical effects. Displayed conduction velocities are those predicted by homogenised models (6) with different choices of boundary conditions (BCs) and averaging volume size, but solved on the original finescale grid to remove the effects of changing the numerical discretisation. All homogenised models perform well, with best performance obtained using the largest averaging volumes to maximise the difference in length scales between finescale features and the homogenised model. Periodic BCs perform best overall, as the medium is of consistent property and hence admits a representative volume element. When obstacles are aligned perpendicular to the direction of propagation, periodic and confined BCs pre-emptively predict conduction block for high proportions of obstruction. Linear BCs instead predict conduction even in the case where the finescale simulation blocks.

In order to further explore the interaction between homogenisation and numerical error, we also separately consider the results of fine-scale models that have their conductivity fields D(x) replaced with the effective fields $D_{\mathrm{eff}}\left(x\right)$ and volume fractions ϕ(x) obtained through the different block homogenisation approaches. This removes the numerical effects of changing the grid spacing and allows homogenisation error to be more directly examined, but of course does not represent a practical use of homogenisation as there is no computational saving. The results of these tests (Fig. 5) present two important conclusions. Firstly, homogenised models perform very well overall, confirming that it is their changed gridsize that produces most of their discrepancy from the equivalent finescale model. Secondly, the overall best-performing boundary conditions now switch to periodic boundary conditions. This is not so surprising, as the tissue slices here have a consistent patterning of obstacles throughout, and it is therefore reasonable to take a representative volume element and treat the medium as periodic [35]. This shows that in the context of the monodomain model (or other models sensitive to the spatial discretisation used to simulate them), the potentially compensatory balance between homogenisation and grid error must be considered in selecting the parameters of the homogenised model.

We also use this scenario to demonstrate the benefits of homogenisation in terms of computational cost savings. Although the finescale model is feasible to simulate in the two-dimensional setting we have considered here, this still required approximately three hours (intelligently terminating the simulation at 110 µs of cardiac activity, that is, after all accessible sites were activated). In contrast, the lowest amount of homogenisation trialled (upscaling from 10 µm to 50 µm) required less than four minutes to simulate cardiac activity over the same period, including both the one-off cost of constructing the homogenised model, and then its simulation (Table 1). The fastest homogenisation level (upscaling to 250 µm) required less than half a minute. These timing tests did leverage the ability to easily parallelise across individual closure subproblems (using six cores), but even without doing this the speed gains are clearly evident.

**Table 1:** Homogenisation represents significant time savings for cardiac electrophysiology. Runtimes for the generation of a single activation map for an example 2D tissue slice of fibrotic tissue (20% of sites marked obstructed at random). As expected, reducing the number of nodes and elements represents a huge reduction in computation time, and the time required to construct homogenised models (solving (9) for each coarse grid element) is minor compared to the time required for simulating on the finescale. Time required to homogenise is non-monotonic with respect to gridsize owing to a balance between the size of each closure problem, and the number of closure problems to be solved.

Mesh spacing	Homogenisation time	Simulation time	Total time	Speedup factor
10 µm	–	10 594.2 s	10 594.2 s	–

50 µm	29.4 s	186.8 s	216.2 s	49.0
100 µm	16.6 s	39.5 s	56.1 s	188.8
250 µm	21.6 s	5.8 s	50.5 s	209.8
500 µm	28.9 s	2.1 s	31.0 s	341.7

### 3.3 Volume averaging allows prediction of source/sink mismatch events

**Fig. 6:**
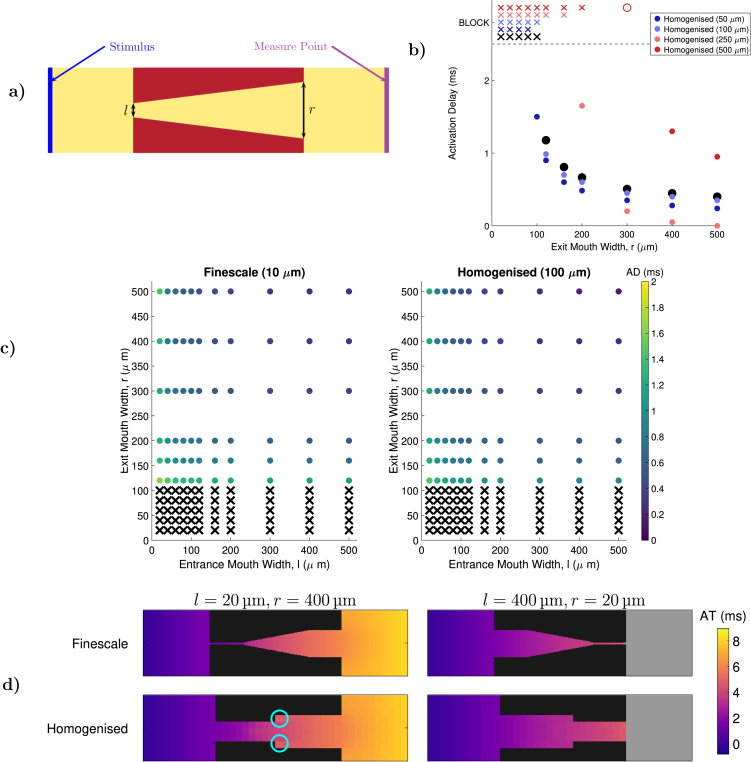
Homogenisation for the capture of source/sink mismatch. (a) The nozzle structures used to evaluate the performance of homogenisation. Activation delay (AD) is measured at the location indicated, and calculated by comparing activation times to those seen when simulating the same domain but with no obstruction present. (b) AD and conduction block as predicted by homogenised models using linear boundary conditions for closure subproblems (9), for a range of exit widths r (l=200µm). Failure to activate the measure point (conduction block) is marked by an × placed at the top of the vertical axis. Good agreement with the finescale model is achieved for homogenised models with grid spacing Δx≤100µm. (c) AD (dot colour) and conduction block (×) in the finescale model, and the best-performing homogenised model using linear boundary conditions (Δx=100µm), for multiple combinations of l and r. Delay is well estimated by the homogenised model, and block is perfectly predicted in all trialled scenarios. (d) Maps of activation time (AT) for two finescale problems (top row) and the equivalent homogenised models (bottom row). Despite changes to the effective channel width in the homogenised models, the timing of activation is well recovered. Noticeable delay in activation is seen where the homogenised model’s larger grid size results in a sudden change in channel width (light blue circles)..

A critical component of the pro-arrhythmic effects of fibrosis is so-called *source/sink mismatch*, in which spatial variation in the amount of excitable tissue can create structures that permit conduction in one direction and not another [2]. Unlike the other homogenised models that have been used to represent the impacts of obstacles in cardiac electrophysiology [19], [20], [21], the explicit representation of the local proportion of conductive tissue (ϕ) in the homogenised models we construct provides them the potential to capture this important electrophysiological dynamic. We examine this potential using a set of small-scale nozzle-like structures (Fig. 6a) that produce a delay in activation, or outright block, when activation reaches the end of the structure and attempts to emerge out into unobstructed tissue. These simulations use an isotropic conductivity tensor with the same baseline conductivity as before, D=3I.

Propagation success is seen to depend predominantly on the width of the exit opening, r, while the width of the entrance, l, is expected to only effect borderline cases. Both widths together control the extent of activation delay (calculated by comparing the activation time at the opposite end of the tissue to the activation time when no obstacle is present). We explore how well our homogenised models predict these dynamics, and highlight the fact that the sizes of the averaging volumes trialled are certainly large enough to obscure the fine detail of the structure, and in most cases alter the effective width of the entrance and exit.

Homogenisation performance depends strongly on both the choice of boundary conditions, and the size of the averaging volume. Homogenisation using periodic or confined boundary conditions was found to mispredict conduction success or failure, regardless of the homogenised model’s new length scale (Figures S2–S3). This is most likely due to the poor handling of diagonal transport seen for these choices in our test case (Fig. 2). The nozzle structure considered here also clearly violates the assumption of periodicity. Linear boundary conditions, however, perform well providing the averaging volume is not made too large (Fig. 6b). In particular, homogenisation by a factor of ten (resulting in a mesh spacing of 100 µm, consistent with high-fidelity anatomic meshes) proved capable of perfectly predicting the success or failure of propagation for all combinations of l and r values trialled (Fig. 6c), and predicted delay accurately in the majority of cases.

Two example activation time maps using this best-performing homogenisation are presented in Fig. 6d. As any averaging volume that contains even a small amount of conductive material will become a non-obstructed element in the homogenised model, this results in a complete loss of the precise shape of the nozzle structure. Nevertheless, the pattern and timing of activation is well recovered. Through its choice of effective tensors and incorporation of the volume fraction, the homogenised model is seen to compensate for this effect. Where the channel is widened artificially by homogenisation, activation noticeably lags (highlights in Fig. 6d) , resulting in additional sink to slow propagation. The homogenised model thereby captures the increasing sink experienced by a wavefront travelling through a widening structure, albeit in a phenomenological fashion as it does not explicitly represent the details of that structure. In the opposite case of a narrowing channel, these regions activate more rapidly than in the centre of the channel, and thus replicate the source-favoured balance for a wavefront moving through a narrowing structure.

### 3.4 Spiral wave anchoring can be predicted by homogenised models

A primary cause of arrhythmia are spiral waves, where cardiac tissue falls into a self-sustaining pattern of continuous re-activation. Depending on both electrophysiological and structural conditions, these spirals may stay fixed rotating about a single region of the tissue or wander about it (with a chance of self-annihilation upon collision with a tissue boundary). Fibrosis can act to stabilise these spiral waves, with even small amounts of diffusely placed obstacles shown to reduce spiral core wander [31]. Larger non-excitable obstacles can also act as fixed locations to which a spiral wave anchors, and is thus more likely to persist [53]. We explore here whether a homogenised model can still produce this latter behaviour, an important component of fibrosis’ pro-arrhythmic effect.

**Fig. 7:**
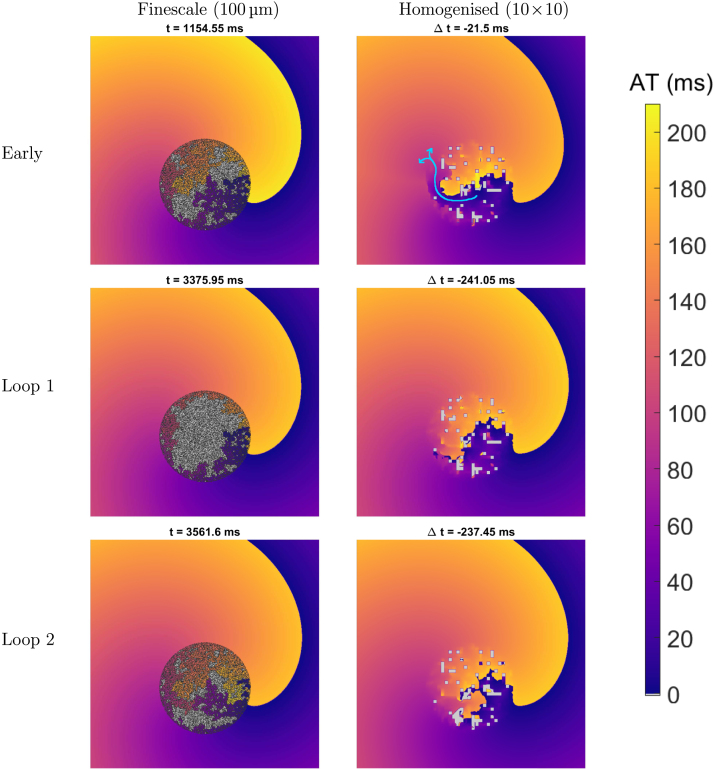
Anchoring of spiral waves in the 10×10 homogenised model. Activation time (AT) maps for the finescale and 10×10 homogenised models in a 6 cm×6 cm slice of tissue. Pictured are an activation before dynamics have stabilised (“early”) and two subsequent activations after stabilisation (“loop 1” and “loop 2”). Light grey sites are those that were not activated in the 210 ms time window, and dark grey sites are those fully occupied by fibrosis. Values of Δt indicate the difference in timing between the finescale and homogenised models for the same activation event. The scar region causes a spiral wave that would otherwise break up to instead anchor and persist indefinitely. The homogenised model successfully predicts this anchoring despite losing the detailed structure of the obstructed region, but slightly underpredicts re-entrant frequency (here 5.45 Hz in finescale versus 5.32 Hz in homogenised). Precise paths of tortuous conduction (and conduction block) are lost in the homogenised model, but the resultant patterns of activation are similar after stabilisation (bottom two rows). Before dynamics stabilise, the over-prediction of conduction due to linear boundary conditions can allow activation to traverse the scar region, resulting in premature activation of the opposing side (light blue arrow in top right panel).

Spiral waves were simulated in two-dimensional, 6 cm × 6 cm slices of isotropic (D=3I) tissue, with a “finescale” grid spacing of Δx=100µm used for reasons of computational cost. Spiral waves were initiated using the common cross field stimulus protocol [54], with a travelling wave initiated at one edge of a two-dimensional slice of tissue, and then a second stimulus triggered in one quadrant of the domain, timed to coincide with the repolarisation front from the first wave. We use the “steep restitution” set of parameter values provided by ten Tusscher et al. [30], which cause spiral waves to break up and devolve into irregular patterns of activation. However, then including a region of scarring represented by 60% of elements being occupied by fibrotic obstruction causes the spiral wave to anchor to the scar’s location. This causes re-entrant activity to be sustained apparently indefinitely without wave breakup. Activation maps corresponding to a rotation of the spiral wave before dynamics completely stabilise, and two subsequent rotations of the spiral wave after stabilisation, are given in Fig. 7.

Inside the scar region, propagation of activation follows tortuous paths of conductive tissue (see left column of Fig. 7, and accompanying supplementary movies). Perfect capture of these very complexly conducting structures with upscaled regions of a single effective conductive property is of course not feasible. However, following their good performance in the previous scenarios considered here, we explore whether such upscaled tensors can at least capture the meaningful effects of the scar region, in particular the stabilisation of spiral wave patterns of re-entry.

We create homogenised models using linear boundary conditions, and averaging volumes of size 10×10 and 25×25 elements. As the base grid in these simulations is already 100 µm, we avoid the numerical effects of significantly larger grid spacings by retaining the base grid but overlaying the tensors obtained by block homogenisation. This approach allows for feasible simulation of sufficient tissue to support spiral waves on the finescale, to which the predictions of homogenised models can be directly compared. We stress, however, that the most practical use case of homogenisation remains incorporating very fine scale (∼ 10 µm) structures into typical cardiac meshes (∼100–250 µm).

Most immediately, the homogenised models are seen to correctly predict the anchoring of spiral waves to a region of obstruction (Figs. 7 and S4). Although the tortuous structures inside the scar region are lost in the homogenisation process, the homogenised models still produce an approximation to the resultant patterns of activation, both inside and especially outside of the scar region. As previously observed, linear BCs tend towards overprediction of conductivity, and so discrepancies between the two models take the form of activations in the homogenised model of locations that do not activate in the finescale. In particular, this can result in premature activation of tissue opposite the scar region in the homogenised model, when excitation spuriously propagates all the way through it (Fig. 7, top right, and also see supplemental movie). A similar effect is almost observed for the 25×25 homogenised model, however in that case conduction blocks when the wave attempts to emerge back into the non-fibrotic tissue (Figure S4, top right).

After many rotations, as the dynamics of the spiral wave re-entry become more similar from rotation to rotation, the agreement between the finescale and homogenised models also improves. The timings with which different sections of the scar activate in the finescale model are then also generally well represented when using upscaled tensors, which capture some of the patterns of conduction block inside the scar and generate near equivalent maps of activation outside the scar. Frequencies of rotation averaged over the last four complete rotations are very similar, 5.45 Hz for the finescale model, 5.31 Hz for the 10×10 homogenised model, and 5.29 Hz for the 25×25 homogenised model. Thus, the upscaled tensors successfully predict both the anchoring of the spiral wave, and the dominant frequency of the resultant arrhythmia. However, the precise timings of each activation event are not matched (values of Δt in Figs. 7 and S4), primarily due to the more significant discrepancies between the two models before dynamics fully stabilise.

Finally, the combination of upscaled tensors but a finescale grid creates an interesting artefact, particularly noticeable in the 25×25 homogenised model (Figure S4, but especially the accompanying movie). Very low conduction in the scar region, but appropriately combined with a small value for the volume fraction, allows “pockets” of excitability to slowly propagate across individual elements of the upscaled grid (the small, isolated regions with distinct activation time in the figure). These might be seen as the homogenised model capturing at least the spirit of slow, tortuous conduction in the scar region, even though the precise paths followed in the finescale model are unavailable.

## Conclusions

Homogenisation has seen only limited use in the modelling of fibrosis in cardiac electrophysiology, despite the technique presenting a natural means for incorporating the effects of sub-mesh-scale obstructions such as cardiac fibrosis into existing heart meshes. The dynamic behaviours seen in electrophysiological models, owing to their highly influential and strongly non-linear reaction terms, do present a significant challenge to prototypical homogenisation, which concerns itself only with the calculation of macroscopic transport properties. However, by careful application of the volume averaging theory for non-conductive obstructions [35], and a thorough consideration of how different choices of closure problem boundary conditions behave in several pernicious cases of interest in this field, we have demonstrated how homogenisation can robustly capture some of the key dynamics through which fibrosis promotes arrhythmia. Specifically, we have demonstrated the potential of the technique to produce homogenised models that accurately capture conduction slowing, especially up to about 30 to 40% obstructed tissue, matching the proportions of collagen that can be seen in typical histological sections of fibrotic tissue [6]. Our homogenised models have also demonstrated successful prediction of source–sink mismatch effects including unidirectional conduction block, and spiral wave anchoring to fibrotic regions.

We have thoroughly investigated the performance of different choices for closure subproblem BCs, both inclusive of, and separate from, the grid error incurred by upscaling in the monodomain model (and bidomain model) [5]. Our results demonstrate linear BCs as the most robust choice in this context, despite their reputation for reduced accuracy in other homogenisation contexts [40]. In addition to being able to correctly predict transport through thin conductive channels (Fig. 2), this choice of BCs has the further benefit that it tends to over-predict conductivity when it does incur homogenisation error, and this error then acts to balance the under-prediction of conductivity that typically comes from increasing the mesh spacing in the monodomain model. Linear BCs also best captured conduction block events due to source–sink mismatch in homogenised models.

The best-performing homogenised models here used reductions in node count by factors of 100 and 625 compared to simulations explicitly resolving finescale detail, representing significant computational speedup. In three dimensions, this speedup is expected to be even more pronounced. Although there is a computational cost associated with solving the closure problems that define effective conductivities in a homogenised model, this is a one-off cost, involves no timestepping, and for the problems considered here was only minor relative to the cost of simulating on the finescale. Where the solution of closure problems threatens to become a bottleneck, they may be solved in parallel or via semi-analytical techniques that further reduce the time required [55].

To demonstrate and quantitatively validate the method, we have used regular, fine-scale (10µm) meshes that captured the spatial scale of fibrotic obstructions to conduction, in two dimensions. As demonstrated through the use of skin around the averaging volume in order to reduce boundary effects, the averaging volume does not need to be the same as the volume over which the closure problem is solved. This allows the approach to extend naturally to irregular meshes, as averaging volumes of any shape may still be embedded in rectangular regions that use a regular finescale grid, at which point the concept of periodicity and any choice of closure problem BCs may be applied. Calculating the proportion of the average volume occupied by collagen that sits on a different grid can be handled via Monte Carlo integration, as described in [22]. These ideas also apply to volumetric meshes in three dimensions, however the once-off cost of solving a closure problem for each element will increase with the larger number of elements in this case. As each closure problem is a steady-state diffusion problem that discretises to become a single linear system solve, and may be solved in parallel, this computational cost is expected to be minor, certainly compared to the cost of simulation on a finescale equivalent of the three-dimensional mesh.

In practice, a modeller will most likely have a coarser-scale (100–250µm) mesh, regular or irregular and in two or three dimensions, on which they wish to simulate cardiac activity in the presence of cardiac fibrosis. Current non-invasive imaging techniques for fibrosis can be used to inform where to place regions of affliction in computational studies [4], [32], and operate at increasingly impressive resolutions [56]. In terms of the placement of finescale collagenous obstructions inside regions of fibrosis, however, this would be achieved in consideration of histological data, or more realistically, using a histologically-informed computational generator of microfibrotic patterns [25]. Studies incorporating macroscopic regions of fibrosis from imaging data into computational models typically seek to capture its net effect, adjusting for example conductivity and electrophysiological properties (for example [56]). Our homogenisation approach serves as a tool for informing these conductivity modifications, providing a quantitative way to determine how different patterns of collagen deposition affect the larger-scale conductivity of afflicted tissue. The primary limitation of homogenisation in this context is of course the elimination of the microscopic structure by the homogenisation process. Although we have demonstrated capture of source/sink mismatch and spiral wave anchorage through proof-of-concept experiments, we have not exhaustively tested whether homogenised models can predict the precise manifestations of these effects across the many different types of obstacle arrangement that may be of interest. The further challenge of capturing re-entries that “live” on the micro-scale [3] has also not been considered here. Additionally, although the method presented here generalises naturally to three dimensions, we have not tested the capture of pro-arrhythmic phenomena in a three-dimensional setting. As including an extra dimension meaningfully changes source/sink balance [57], the prediction of conduction block in homogenised models might also be affected by a third spatial dimension.

Overall, homogenisation has been shown here to perform better than might be expected, considering the reaction-dominated dynamics of excitation propagation and the complex effects of spatial heterogeneity on these dynamics. Used with care, and making use of the extensions to the base technique we have presented such as explicitly incorporating heterogeneity in the volume of excitable tissue, homogenised monodomain models have proven capable of predicting several key phenomena of interest in cardiac electrophysiology. The technique should also be applicable to travelling wave dynamics in other contexts, for example Ca^2＋^ [58] or cyclic adenosine monophosphate [59] signalling, where cytoskeletal obstructions motivate homogenisation [60]. Indeed, following the result that discrepancies in homogenised models owed mostly to the numerical consequences of changing grid spacing, we suspect the approach we present here would perform even better in models where waves are less steep-fronted.

## CRediT authorship contribution statement

**Brodie A.J. Lawson:** Conceptualization, Methodology, Software, Investigation, Formal analysis, Writing – original draft, Writing – review & editing. **Rodrigo Weber dos Santos:** Conceptualization, Methodology, Writing – review & editing. **Ian W. Turner:** Methodology, Formal analysis, Writing – review & editing. **Alfonso Bueno-Orovio:** Investigation, Writing – review & editing. **Pamela Burrage:** Writing – review & editing. **Kevin Burrage:** Conceptualization, Investigation, Writing – review & editing, Supervision.

## Declaration of Competing Interest

The authors declare that they have no known competing financial interests or personal relationships that could have appeared to influence the work reported in this paper.

## Appendix A Control volume finite element method for rectangular grid

Here we present the precise form of the control volume finite element scheme for a two-dimensional regular rectangular grid, as used in this work. For such a grid, bilinear interpolation is suitable for the interpolants in (15). If we take the bottom left corner of an element as the origin, such an interpolant takes the form (A.1)

$$V_{i}=f\left(V_{dl,i},V_{ul,i},V_{dr,i},V_{ur,i},x,y\right)=V_{dl,i}+\left(V_{ul,i}-V_{dl,i}\right)\;\;\frac{x}{\Delta x}+\left(V_{dr,i}-V_{dl,i}\right)\;\;\frac{y}{\Delta y}+\left(V_{ur,i}-V_{ul,i}-V_{dr,i}+V_{dl,i}\right)\;\;\frac{x\;y}{\Delta x\;\Delta y},$$

with $\left(V_{dl,i},V_{ul,i},V_{dr,i},V_{ur,i}\right)$ the vertices of the ith mesh element, as pictured in Figure S5. Via appropriate use of the transformations $x^{\prime }=\Delta x-x$ and $y^{\prime }=\Delta y-y$, we may treat all four elements associated with a control volume as having the node at the control volume’s centre as the origin (bottom left corner). This motivates labelling the vertices of each element in terms of how they relate to the control volume node, as demonstrated in Figure S5. Briefly, $V_{p}$ refers to the control volume’s central node, $V_{x,i}$ refers to the vertex of the ith element that is an x translation away from the central node, and similarly for $V_{y,i}$ and $V_{xy,i}$. Using this re-labelling, the contribution of each portion of the control volume now has the same form, so that $m_{i}$ and $k_{i}$ in Eq. (16) become

$$m_{i}=\int_{\Omega_{\mathrm{CV},i}}V_{i}\;d\Omega_{\mathrm{CV},i}=\int_{0}^{\Delta x/2}\int_{0}^{\Delta y/2}f\left(V_{p,i},V_{x,i},V_{y,i},V_{xy,i},x,y\right)\;dxdy=\Delta x\Delta y\left(\frac{9}{64}V_{p,i}+\frac{3}{64}V_{x,i}+\frac{3}{64}V_{y,i}+\frac{1}{64}V_{xy,i}\right)$$

$$k_{i}=\int_{\sigma_{\mathrm{CV},i}}D\nabla V_{i}\cdot \overset{ˆ}{n}\;d\sigma_{\mathrm{CV},i}=\int_{0}^{\Delta x/2}\left[D_{11}\left(V_{x,i}-V_{p,i}+\left(V_{xy,i}-V_{y,i}\right)\frac{\Delta y}{2}\right)+D_{12}\left(\left(V_{y,i}-V_{p,i}\right)\left(\Delta x-x\right)+\left(V_{xy,i}-V_{x,i}\right)x\right)\right]\;dx$$

$$\;+\int_{0}^{\Delta y/2}\left[D_{22}\left(V_{y,i}-V_{p,i}+\left(V_{xy,i}-V_{x,i}\right)\frac{\Delta x}{2}\right)+D_{12}\left(\left(V_{x,i}-V_{p,i}\right)\left(\Delta y-y\right)+\left(V_{xy,i}-V_{y,i}\right)y\right)\right]\;dy=\Delta x\Delta y[\frac{D_{11}}{\Delta x^{2}}\left(\frac{3}{8}\left(V_{x,i}-V_{p,i}\right)+\frac{1}{8}\left(V_{xy,i}-V_{y,i}\right)\right)+\frac{D_{22}}{\Delta y^{2}}\left(\frac{3}{8}\left(V_{y,i}-V_{p,i}\right)+\frac{1}{8}\left(V_{xy,i}-V_{x,i}\right)\right).$$

$$\;\;.+\frac{1}{2}\frac{D_{12}}{\Delta x\Delta y}\left(V_{xy,i}-V_{p,i}\right)].$$

In the case of constant D and ϕ across the domain, the resulting mass and stiffness matrices then take the stencil form [61]

$$M=\phi \left(\begin{array}{ccc} \frac{1}{64} & \frac{3}{32} & \frac{1}{64}\\ \frac{3}{32} & \frac{9}{16} & \frac{3}{32}\\ \frac{1}{64} & \frac{3}{32} & \frac{1}{64} \end{array}\right)\;\;\;\;K=\phi \left(\begin{array}{ccc} \frac{1}{8}\left(\frac{D_{11}}{\Delta x^{2}}+\frac{D_{22}}{\Delta y^{2}}\right)-\frac{1}{2}\frac{D_{12}}{\Delta x\Delta y} & \frac{3}{4}\frac{D_{22}}{\Delta y^{2}}-\frac{1}{4}\frac{D_{11}}{\Delta x^{2}} & \frac{1}{8}\left(\frac{D_{11}}{\Delta x^{2}}+\frac{D_{22}}{\Delta y^{2}}\right)+\frac{1}{2}\frac{D_{12}}{\Delta x\Delta y}\\ \frac{3}{4}\frac{D_{11}}{\Delta x^{2}}-\frac{1}{4}\frac{D_{22}}{\Delta y^{2}} & -\frac{3}{2}\left(\frac{D_{11}}{\Delta x^{2}}+\frac{D_{22}}{\Delta y^{2}}\right) & \frac{3}{4}\frac{D_{11}}{\Delta x^{2}}-\frac{1}{4}\frac{D_{22}}{\Delta y^{2}}\\ \frac{1}{8}\left(\frac{D_{11}}{\Delta x^{2}}+\frac{D_{22}}{\Delta y^{2}}\right)+\frac{1}{2}\frac{D_{12}}{\Delta x\Delta y} & \frac{3}{4}\frac{D_{22}}{\Delta y^{2}}-\frac{1}{4}\frac{D_{11}}{\Delta x^{2}} & \frac{1}{8}\left(\frac{D_{11}}{\Delta x^{2}}+\frac{D_{22}}{\Delta y^{2}}\right)-\frac{1}{2}\frac{D_{12}}{\Delta x\Delta y} \end{array}\right).$$

These are slightly different to those derived using a typical finite element method, and thanks to the control volume formulation, result in a conservative numerical scheme. For reference, when D=DI and Δx=Δy=Δ, the stiffness matrix becomes

$$K=\phi \frac{D}{\Delta^{2}}\left(\begin{array}{ccc} \frac{1}{4} & \frac{1}{2} & \frac{1}{4}\\ \frac{1}{2} & -3 & \frac{1}{2}\\ \frac{1}{4} & \frac{1}{2} & \frac{1}{4} \end{array}\right).$$

## Appendix B Supplementary data

Video S1 Supplementary material: Additional figures and movies referenced in the main text.

Video S3

MMC S1

## Footnotes

2 Although it may seem as though this periodicity argument could break when a segment of $\sigma_{b}$ has no matching component on the opposite side of Ω to cancel it out (because the opposite side is occluded), these segments are in fact then segments of $\sigma_{m}$ due to the assumption of periodic substructure, and thus sent to zero by the occlusion boundary condition instead.
