# Supplementary material for: Homogenisation for the monodomain model in the presence of microscopic fibrotic structures
Source: Commun Nonlinear Sci Numer Simul. 2023 Jan;116:None. doi: 10.1016/j.cnsns.2022.106794 (PMC10124103; doi:10.1016/j.cnsns.2022.106794)
Supplement: MMC S1 [file mmc4.pdf]

Appendix B. Supplementary Figures

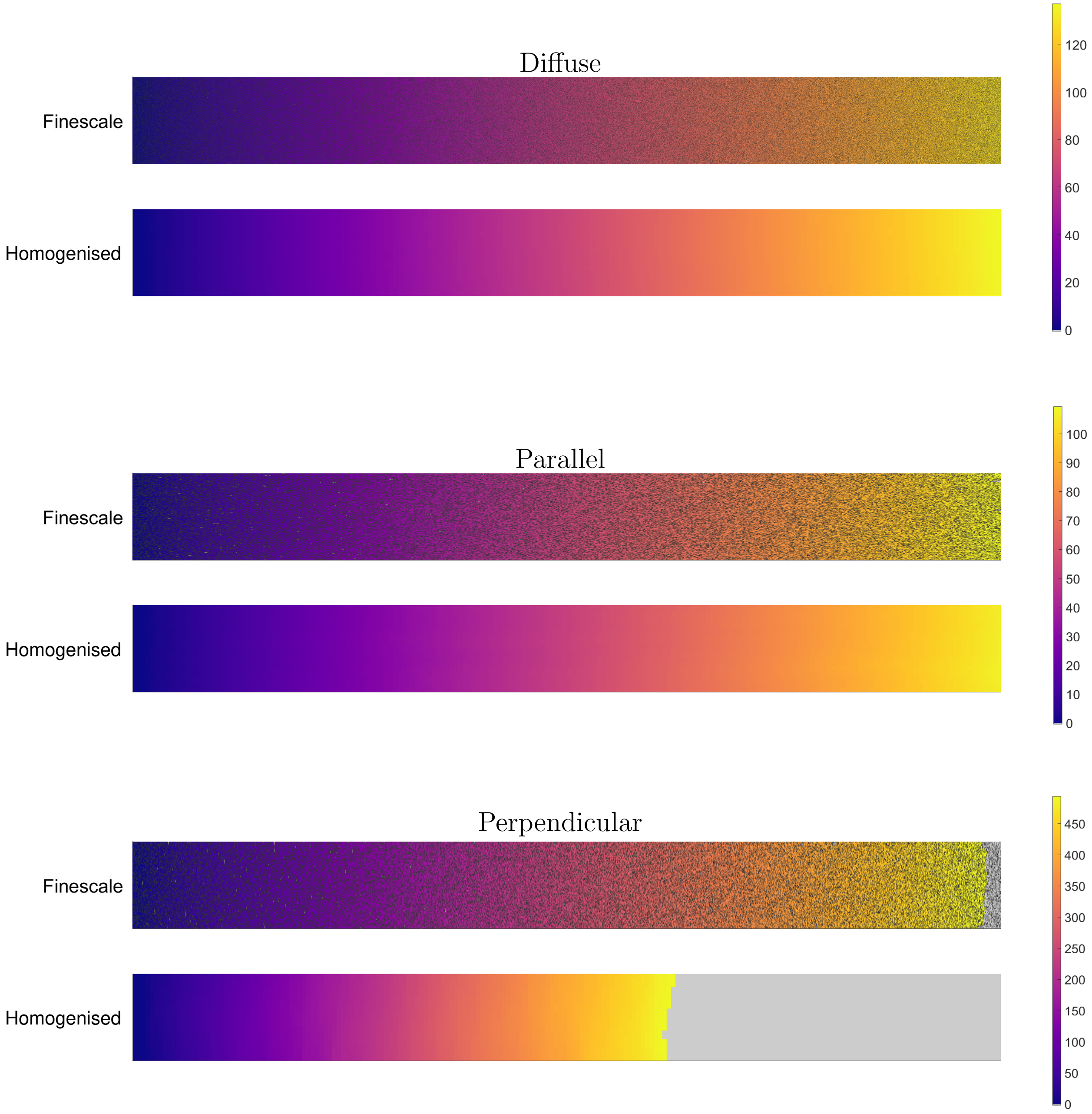

Figure S1: **Example activation maps for the activation delay test problem.** Activation time maps (in ms) for finescale and homogenised ($\Delta x = 250\,\mu\text{m}$) models, for different patterns of fibrotic obstruction. Even for this moderate level of obstruction (35%), activation dynamics are essentially those of a planar wave, with approximately constant velocity. The same activation dynamics are observed in the homogenised models (visualised are for $\Delta x = 250\,\mu\text{m}$), and as such the error incurred by upscaling manifests as inaccuracies in conduction velocity. In the most challenging case (perpendicular strands of fibrosis), the wave is no longer perfectly planar, and its shape is not necessarily consistent between the finescale and homogenised models (even at the same location). However, error due to upscaling remains primarily due to underprediction of velocity.

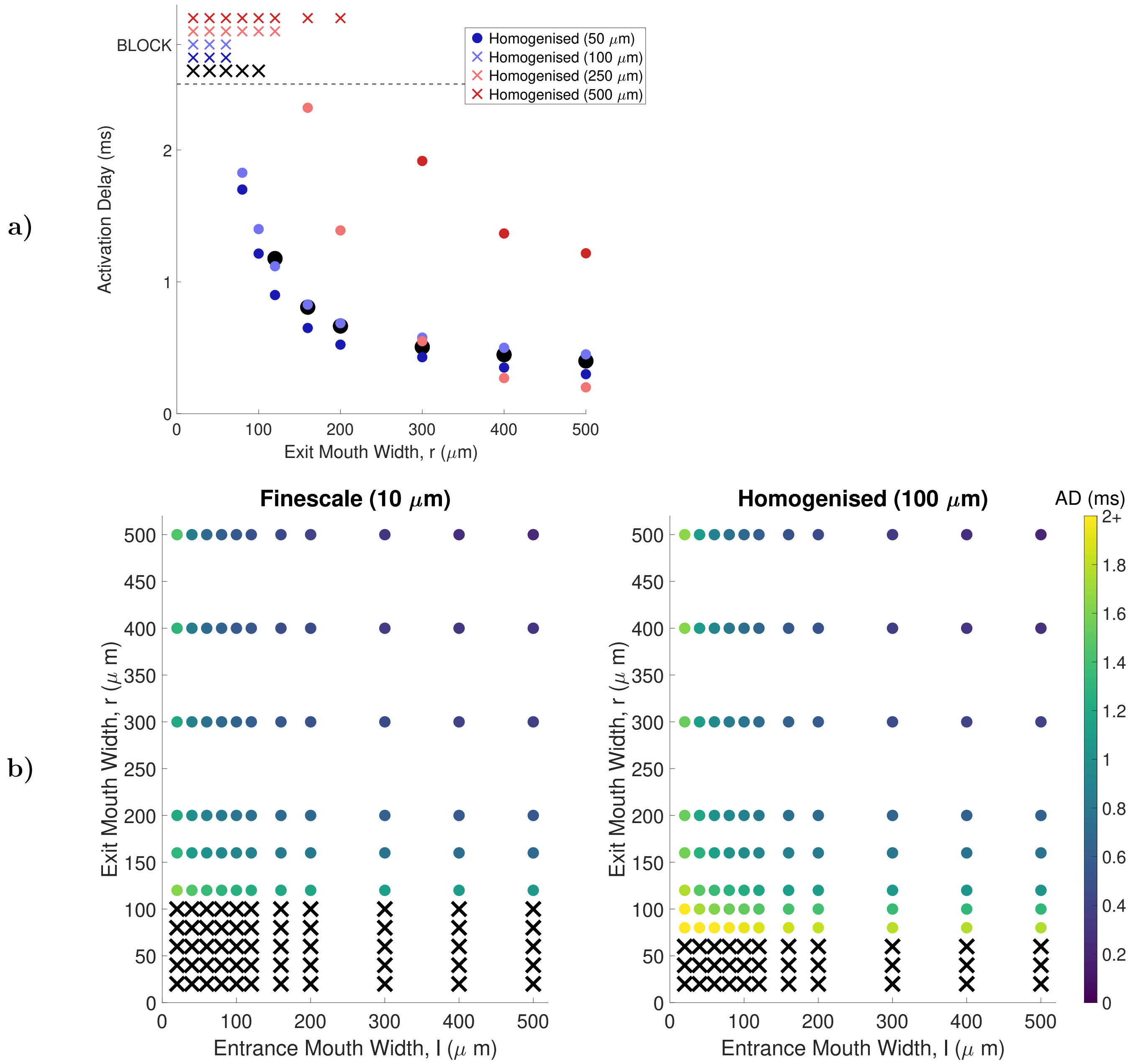

Figure S2: **The performance of confined boundary conditions to capture the effects of source/sink mismatch. a)** Activation delay (AD) and conduction block as predicted by confined boundary condition homogenised models of different characteristic lengths, for a range of exit widths r ($l = 200\,\mu\text{m}$). When conduction blocks completely, this is indicated by an $\times$ placed at the highest part of the vertical axis. Large characteristic lengths for homogenised models tend towards overestimation of AD and block, while the smaller characteristic lengths result in underestimation. **b)** AD (coloured dots) and conduction block ($\times$) as observed in the finescale model, and predicted by the best-performing homogenised model with confined boundary conditions ($\Delta x = 100\,\mu\text{m}$). Delay in the finescale model is generally well predicted, but the rapid transition from low AD to block ($r \sim 100\,\mu\text{m}$ in the finescale model) is predicted poorly by even this best-performing homogenised model, indicating a failure of confined boundary conditions.

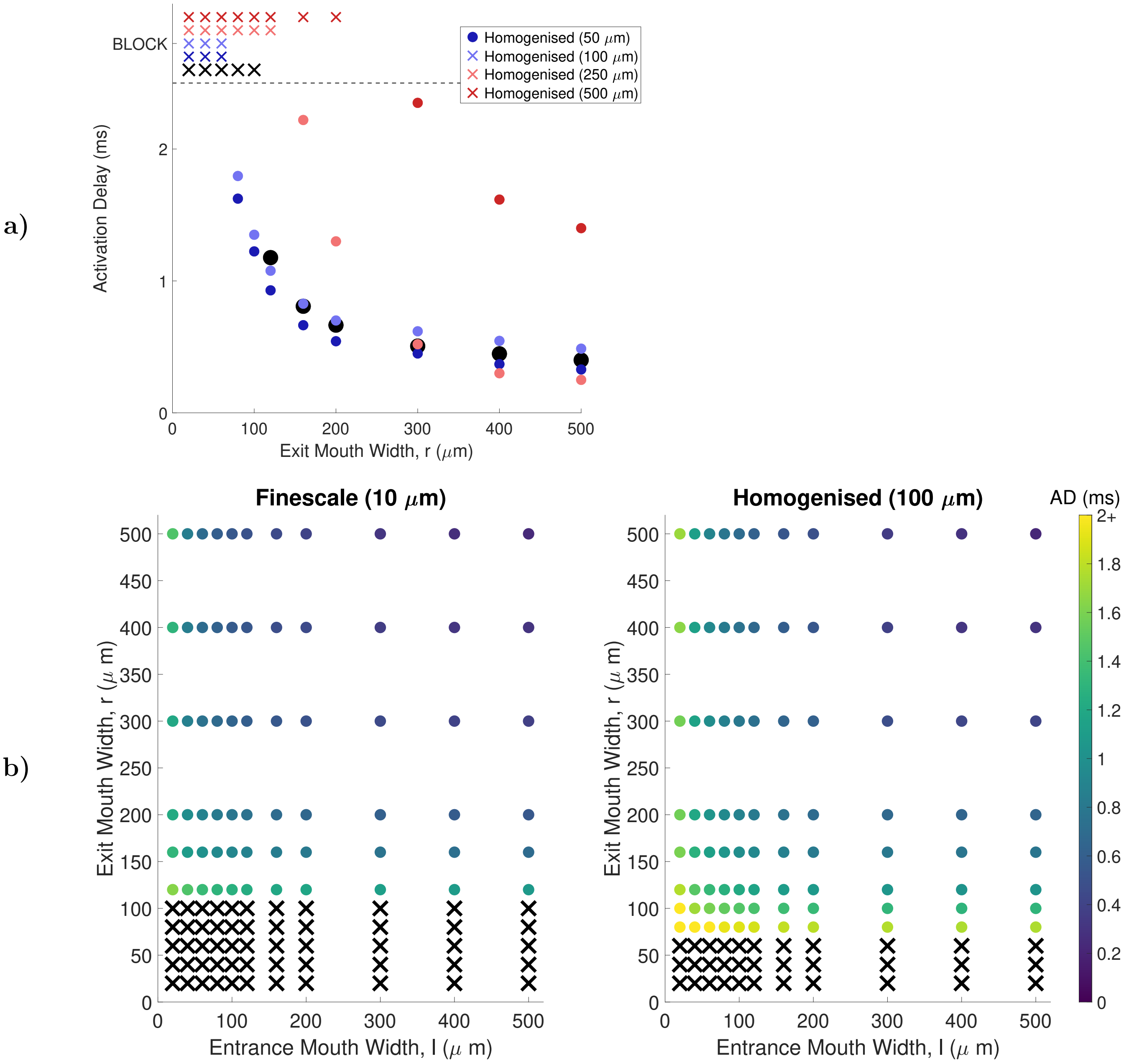

Figure S3: **The performance of periodic boundary conditions to capture the effects of source/sink mismatch.** **a)** Activation delay (AD) and conduction block as predicted by homogenised models of different characteristic lengths, for a range of exit widths r ($l = 200\,\mu\text{m}$). When conduction blocks completely, this is indicated by an $\times$ placed at the highest part of the vertical axis. Large characteristic lengths for homogenised models tend towards overestimation of AD and block, while the smaller characteristic lengths result in underestimation. **b)** AD (coloured dots) and conduction block ($\times$) as observed in the finescale model, and predicted by the best-performing homogenised model with periodic boundary conditions ($\Delta x = 100\,\mu\text{m}$). Delay in the finescale model is generally well predicted, but the rapid transition from low AD to block ($r \sim 100\,\mu\text{m}$ in the finescale model) is predicted poorly by even this best-performing homogenised model, indicating a failure of periodic boundary conditions.

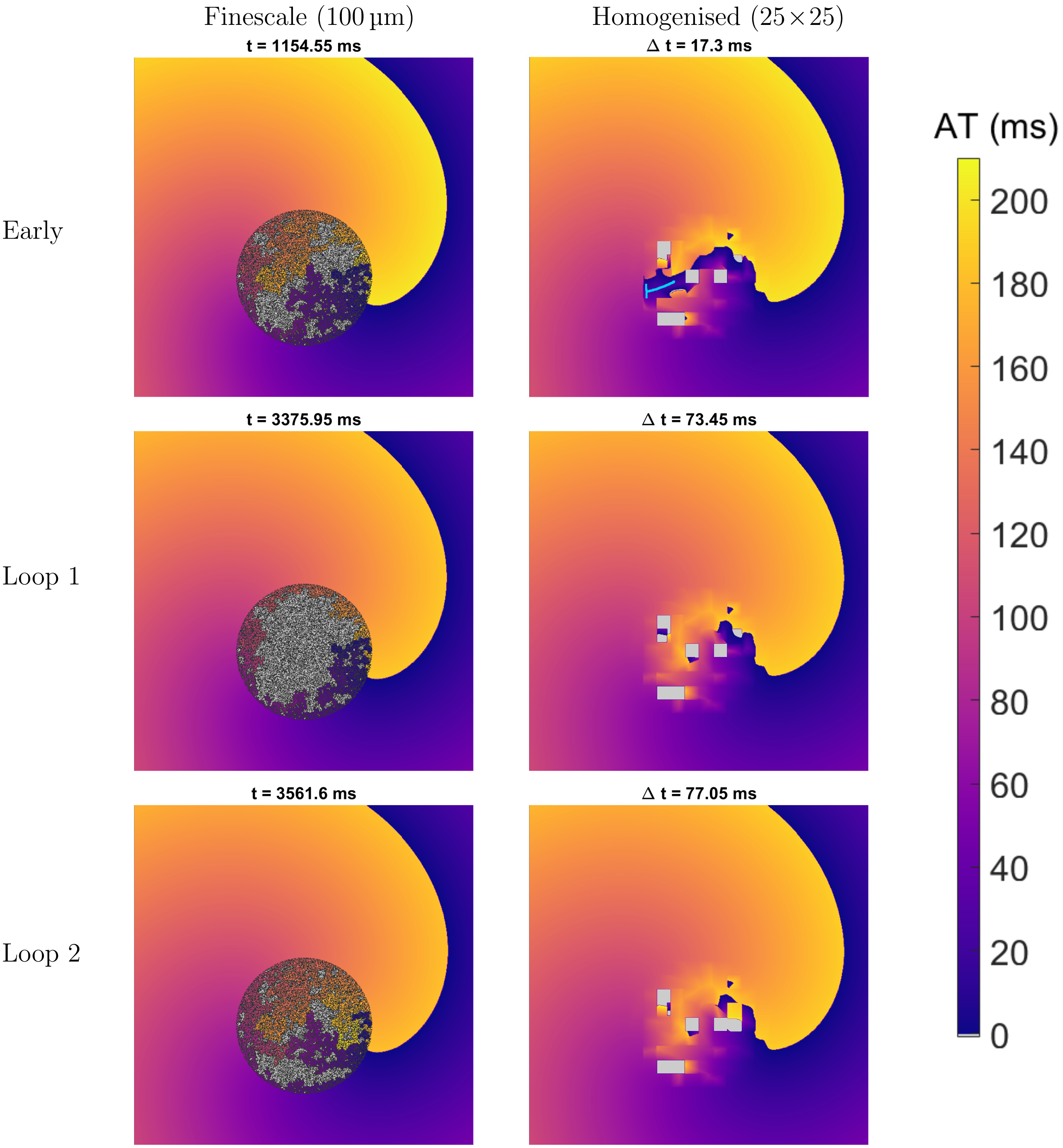

Figure S4: **Anchoring of spiral waves in the 25×25 homogenised model.** Activation time (AT) maps in a 6 cm×6 cm slice of tissue for a spiral wave re-entry stabilised by a region of scarring. Pictured are an activation event before dynamics have stabilised ("early") and two subsequent activations after stabilisation ("loop 1" and "loop 2"), for both finescale (left column) and 25× homogenised (right column) models. Light grey sites are those that were not activated in the 210 ms time window, and dark grey sites are those fully occupied by fibrosis. Values of Δt indicate the difference in timing between the finescale and homogenised models for the same activation event. The homogenised model successfully predicts spiral wave anchoring, but slightly underpredicts re-entrant frequency (5.29 Hz in homogenised versus 5.41 Hz in finescale). Precise paths of tortuous conduction (and conduction block) are lost in the homogenised model, but after dynamics stabilise (bottom two rows), the resultant patterns of activation are similar. Before dynamics stabilise, activation traverses the scar region, but blocks upon attempt to invade back into non-fibrotic tissue (light blue in top right panel).

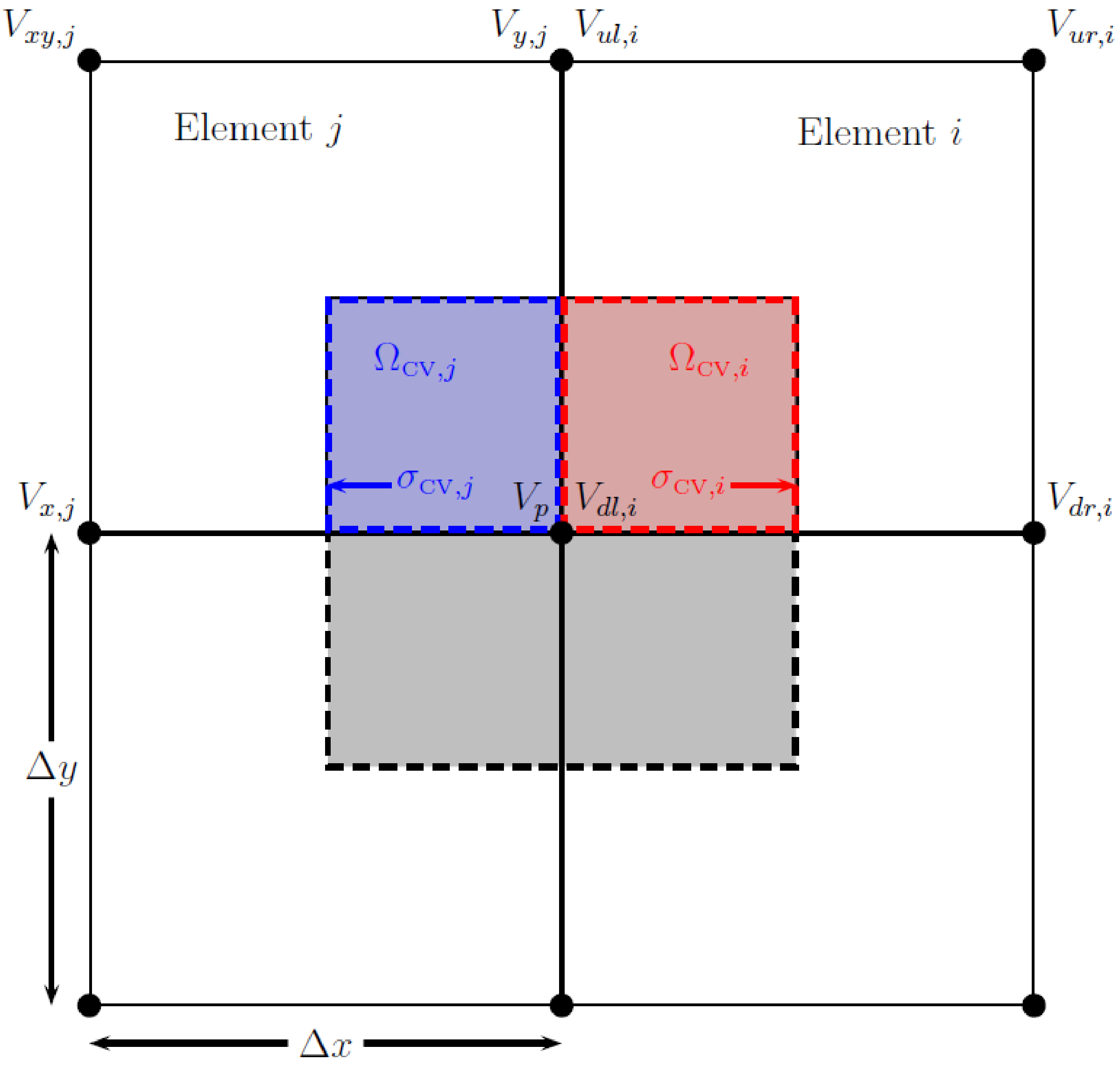

Figure S5: An example control volume for a regular rectangular 2D grid (shaded), and the notation used in describing the control volume finite element scheme. The top two elements demonstrate the two different types of notation used for nodes. The top left element shows the notation based on position relative to the current control volume, for example V_p for the current control volume's node, and $V_{x,j}$ for the vertex of the j-th element that is an x translation away from this node. The top right element shows the notation based solely on the indexed element, for example $V_{dl,i}$ for the down-left node of the i-th mesh element. To highlight the connection between the two, note that for the pictured control volume, $V_{x,i}$ refers to $V_{dr,i}$.
